# Genetically predicted 486 blood metabolites concerning risk of systemic lupus erythematosus: a Mendelian randomization study

**DOI:** 10.1038/s41598-023-49233-8

**Published:** 2023-12-18

**Authors:** Li Zhao, Ruonan Wu, Zewen Wu, Xinling Liu, Jingxuan Li, Liyun Zhang, Shuqiu Zhang

**Affiliations:** 1 School of Pharmacy, Shanxi Medical University, Taiyuan, 030600 China; 2 School of Public Health, Shanxi Medical University, Taiyuan, 030001 China; 3 Shanxi Bethune Hospital, Shanxi Academy of Medical Sciences, Tongji Shanxi Hospital, Third Hospital of Shanxi Medical University, Taiyuan, 030032 China; 4 Shanxi University of Chinese Medicine, Jinzhong, 030619 Shanxi China

**Keywords:** Immunology, Medical research, Pathogenesis, Rheumatology, Risk factors

## Abstract

Metabolic abnormalities constitute a significant characteristic of systemic lupus erythematosus (SLE). We utilised a two-sample Mendelian randomisation (MR) study to evaluate the potential causal association between 486 blood metabolites and SLE. Exposure data at the metabolite level were extracted from 7824 European Genome-wide association studies (GWAS). Preliminary analysis utilised SLE GWAS data from FinnGen. The primary method for causal analysis relied on random inverse variance weighting (IVW). To ensure robustness, sensitivity analyses included the Cochran Q test, MR-Egger intercept test, MR-PRESSO, and leave-one-out analysis. Steiger testing and linkage disequilibrium score regression were employed to validate the identified metabolites. This study identified 12 metabolites, comprising six known chemical structures: 1,5-anhydroglucitol(1,5-AG) [odds ratio (OR) = 0.100, 95% confidence interval (CI): 0.015–0.773, P = 0.027), gamma-glutamylthreonine (OR = 0.077, 95% CI: 0.010–0.574, P = 0.012), 5-dodecenoate(12:1n7) (OR = 0.205, 95% CI: 0.061–0.685, P = 0.010), linoleoylglycerophosphoethanolamine * (OR = 0.159, 95% CI: 0.027–0.933, P = 0.044), erythrose (OR = 88.331,95% CI:1.098–63.214, P = 0.040) and 1-, adrenate (22:4n6) (OR = 9.876, 95% CI: 1.753–55.639, P = 0.001)]. Additionally, we found associations between SLE and six unknown chemical structures: X-06351 (OR = 0.071, 95% CI: 0.006–0.817, P = 0.034), X-10810 (OR = 4.268 95% CI: 1.260–14.459, P = 0.020), X-11412 (OR = 5.418 95% CI: 1.068–27.487, P = 0.041), X-11905 (OR = 0.551, 95%CI: 0.304–0.997, P = 0.049), X-12038 (OR = 0.178 95%CI: 0.032–0.988, P = 0.045), X-12217 (OR = 0.174 95%CI: 0.044–0.680, P = 0.014). This study offers evidence supporting a causal relationship between SLE and 12 circulating metabolites, six of which have known chemical structures and six that remain unidentified. These findings introduce a new perspective for further exploration of SLE mechanisms.

## Introduction

Systemic lupus erythematosus (SLE) is a chronic autoimmune disease affecting multiple organs, yet its precise pathophysiology and specific biomarkers remain largely unknown^1^. This underscores the crucial need to prioritise improved preventive and screening measures for SLE. While prior research has hinted at potential risk factors like gut microbiota^2^ and cytokines^3^, studies focusing on metabolic changes in SLE are relatively scarce.

In recent years, metabolomics has gained significant prominence within systems biology, offering a novel lens to unveil the underlying mechanisms of diseases. Notably, metabolomics has played a pivotal role in identifying and analysing altered metabolites and metabolic pathways, offering valuable insights into the intricate biological mechanisms associated with various diseases, including SLE^4,5^. In the domain of autoimmune diseases, metabolomics holds promise in identifying useful biomarkers^6,7^. Additionally, immune metabolism has emerged as a promising avenue, potentially modulating the differentiation and function of immune cells and offering therapeutic possibilities. Advancements in mass spectrometry-based metabolic flux analysis technology have furthered our understanding of the metabolic profiles of patients with SLE in serum/plasma^8^.

Furthermore, targeted regulation of metabolites holds significant potential for SLE treatment. For instance, Omega-3 fatty acids, specifically eicosapentaenoic acid (EPA) and docosahexaenoic acid (DHA) found in oily fish and fish oil supplements, have been extensively studied. Previous research in cell culture and animal models has showcased their potential to diminish pro-inflammatory cytokine production such as tumour necrosis factor (TNF)-α and interleukin (IL)-1β while elevating the concentrations of the anti-inflammatory cytokine IL-10^9^. These effects have been observed in various conditions, including rheumatoid arthritis, colitis and asthma^10^. Notably, fish oil and its derivatives containing EPA and DHA have been utilised in clinical settings to treat rheumatoid arthritis^11–13^. Hence, the targeted modulation of metabolism, such as through regulatory substitutions with Omega-3 fatty acids, presents promise as a therapeutic strategy for managing SLE.

Exploring metabolites associated with the onset and progression of SLE not only holds significance for early screening and prevention but also carries crucial importance in understanding the biological mechanisms underpinning SLE treatment. However, the causal relationship between metabolites and SLE remains uncertain due to a lack of prospective studies examining metabolites and SLE. Traditional observational studies are limited by design constraints, including changes in patient lifestyle post-SLE diagnosis or alterations in metabolic substances induced by long-term medication use, contributing to an unclear causal relationship between metabolites and SLE. Randomised controlled trials (RCTs) are considered the gold standard in establishing causal effects but pose challenges in this context, making it difficult to derive definitive conclusions regarding the causal relationship between metabolites and SLE.

In the absence of RCTs, Mendelian randomisation (MR) has emerged as a compelling approach to explore the causal relationship between the exposure of interest and its outcomes^14^. MR utilises exposure-related single nucleotide polymorphisms (SNPs) as instrumental variables (IVs) to assess the causal effects of genetic proxy exposures on outcomes^15^. This approach mimics RCTs by randomly assigning genetic variants (SNPs) to offspring during conception, reducing confounding factors, such as gender and age, that might bias causal effects. Additionally, MR minimises the likelihood of reverse causality since the genotype is determined before disease onset^16^.

Given the limited comprehension of the causal association between blood metabolites and SLE, further investigations in this domain are warranted. This study employs MR analysis, utilising aggregated data from genome-wide association studies (GWAS), to comprehensively examine the potential causal involvement of 486 blood metabolites in the development of SLE.

## Methods and materials

### GWAS data for 486 blood metabolites and SLE

Genetic data for 486 metabolites, involving 2,163,597 associated SNPs, were accessed from the metabolomics GWAS server (https://metabolomics.helmholtz-muenchen.de/gwas/)^17^. Detailed names of the 486 metabolites, denoted as X for unknown chemical properties, are listed in Table S1.

The SLE GWAS dataset, obtained from FinnGen (https://www.finngen.fi/en), encompassed 538 cases and 213,145 controls, providing a substantial sample size for analysis (Table 1).

**Table 1:** Details of the GWAS included in the Mendelian randomization.

	Trait	Data sources	Population	N case	N control	Websource
Exposure	Systemic lupus erythematosus	FinnGen	European	538	2,13,145	https://www.finngen.fi/en
Outcome	Metabolites	Metabolomics GWAS server	European	7824	N	https://metabolomics.helmholtz-muenchen.de/gwas/

### Instrumental variable (IV) selection

**Figure 1:**
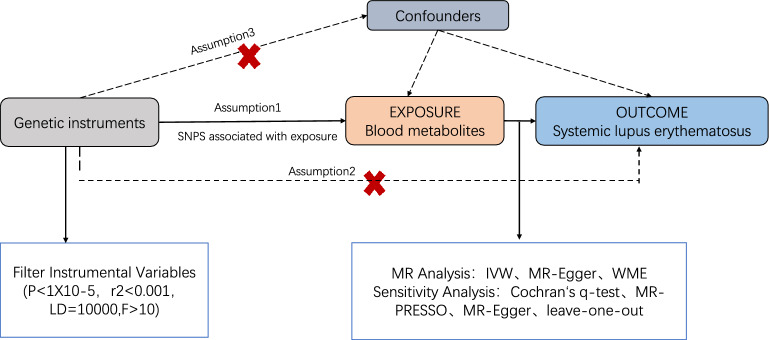
Workflow of the MR analysis.

MR plays a vital for inferring causal relationships between traits. It utilises genetic variation as instrumental variables (IVs) from GWAS data to infer the causal relationship between exposure and outcome. In this study, blood metabolites and SLE were considered the exposure and outcome, respectively. Selected IVs adhered to three key assumptions^18^: (1) genetic variation associated with the exposure; (2) No confounders exist between genetic variation and the exposure-outcome association; (3) genetic variation does not influence the outcome except through its association with the exposure factor. A visual representation of the study's overview is depicted in Fig. 1. To fulfil these criteria, SNPs were utilised as IVs, setting a significance threshold of a P < 1 × 10^–5^ to exclude less significant SNPs and eliminate highly correlated ones for independence^19^. Furthermore, to ensure the independence of SNPs and avoid linkage disequilibrium (LD) bia^20^, we set an LD threshold of r^2^ < 0.001 and a distance of 10,000 kb. The PhenoScanner, (http://www.phenoscanner.medschl.cam.ac.uk/) online tool assessed whether these SNPs were associated with confounding factors in SLE.

### Statistical analysis

The IVW method, widely used in MR research for its robustness^21–23^, was employed in the fixed-effects model and the random-effects model to reduce bias due to heterogeneity. All SNPs included in the IVW method adhered to the three assumptions of IV selection, especially the exclusivity assumption, which requires that genetic variation affects the outcome only through the exposure factors in the study. Despite efforts to exclude confounding SNPs, generic pleiotropy could have causal effect estimates. Therefore, MR-Egger regression and Weighted Median Estimator (WME) methods were used to test the stability of the results. MR-Egger regression modifies the IVW method to test for pleiotropy as well as correct pleiotropy bias. However, the MR-Egger regression method is less statistically valid for causal estimation, with some studies pointing out that the MR-Egger regression method can only be used as a sensitivity analysis to test whether it violates the core assumptions of the instrumental variables and not as an alternative to the IVW method. Conversely, the WME method gives consistent results considering that some of the genetic variations in the analyses are not valid IVs.

To evaluate the strength of IVs, an F-statistic was employed, using the formula F = R2 × (n − k − 1)/[(1 − R2) × k]. In this equation, R2 represents the genetic variance explained by the sample size, n denotes the sample size and k represents the number of SNPs present in the sample^24^. An F-statistic above 10 indicated no significant weak instrument bias. Conversely, SNPs with F-statistics below this threshold were considered weak instruments and excluded from the analysis. Final MR analysis was performed again after eliminating IVs not meeting this criterion, with reliable IVW method results when no heterogeneity or pleiotropy evidence existed^25^.

### Sensitivity analysis

Sensitivity analyses encompassed heterogeneity and multiplicity tests. Differences between the included studies, such as different gene annotation and analysis platforms or different inclusion and exclusion criteria, might contribute to heterogeneity in our results. In this study, Cochran's Q test assessed heterogeneity in IVW and MR-Egger regression methods, with P > 0.05 indicating no statistically significant effect on the study results. As the intercept term in MR-Egger regression approaches zero, the magnitude of horizontal pleiotropy becomes smaller. Notably, horizontal pleiotropy did not exist if the test for horizontal pleiotropy resulted in P > 0.05. MR-PRESSO outlier test identified outlier SNPs affecting overall results. A ‘leave-one-out’ analysis removed one SNP at a time to assess its impact, visually represented through forest plots for result stability. Furthermore, MR Steiger tests verified causal direction.

## Results

**Figure 2:**
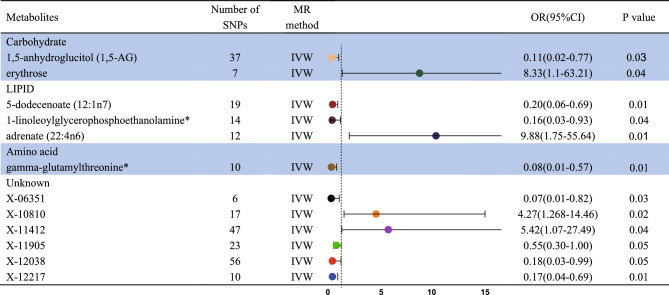
Significant results for the IVW analysis.

**Figure 3:**
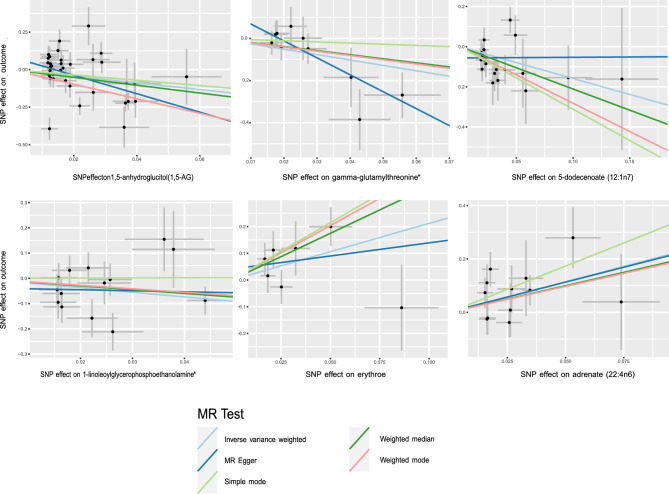
Scatter plots of the MR analysis.

**Figure 4:**
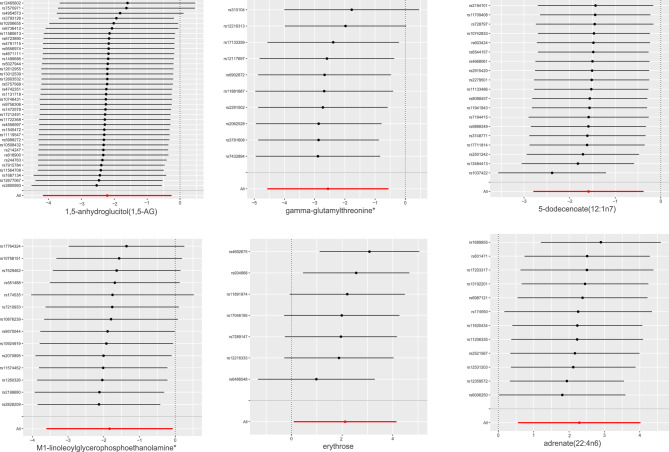
Leave-one-out results.

Following strict IV quality control, the MR Study encompassed 486 metabolites. To ensure accuracy, LD analysis was performed to eliminate any potential chain ambiguity. Additionally, an extensive search through the PhenoScanner database aimed to identify established SLE risk factors yielded no relevant findings (Table S2). Throughout the screening process, special attention was dedicated to removing palindromic sequences to avert potential strand ambiguity, ensuring the reliability of selected IVs. Ultimately, 10,541 SNPs were identified as IVs, constituting a comprehensive set of genetic variants associated with 486 metabolites. The F-statistics of these IVs exceeded 10, indicating their robustness and power. Detailed information about the IVs can be found in Table S3. MR analysis results, including the F-statistic, are visually represented in Table S4. Subsequently, an IVW analysis identified 12 metabolites potentially casually linked to SLE, with six known and unknown compounds (Fig. 2). These findings shed light on the association between metabolites and SLE, providing valuable insights into potential underlying mechanisms. Noteworthy metabolites include 1,5-anhydroglucitol (1,5-AG) (Odds Ratio (OR) = 0.100, 95% Confidence Interval (CI): 0.015–0.773, P = 0.270), gamma-glutamylthreonine (OR = 0.077, 95% CI: 0.010–0.574, P = 0.012), 5-dodecenoate(12:1n7) (OR = 0.205, 95% CI: 0.061–0.685, P = 0.010), linoleoylglycerophosphoethanolamine * (OR = 0.159, 95% CI: 0.027–0.933, P = 0.044), erythrose (OR = 88.331, 95% CI:1.098–63.214, P = 0.040), 1-adrenate (22:4n6) (OR = 9.876, 95% CI: 1.753–55.639, P = 0.001), X-06351 (OR = 0.071, 95% CI: 0.006–0.817, P = 0.034), X-10810 (OR = 4.268, 95% CI: 1.260–14.459, P = 0.200), X-11412 (OR = 5.418 95% CI: 1.068–27.487, P = 0.041), X-11905 (OR = 0.551, 95% CI: 0.304–0.997 P = 0.049), X-12038 (OR = 0.178, 95% CI: 0.032–0.988, P = 0.0448) and X-12217 (OR = 0.174, 95% CI: 0.044–0.680, P = 0.0143). Table 2 presents the results of the sensitivity analyses. Cochran's Q test, assessing potential heterogeneity, specifically the metabolites under investigation, revealed no significant heterogeneity among the IVs. Additionally, MR-PRESSO revealed no outliers, and MR-Egger's intercept analysis suggested an absence of horizontal pleiotropy (Fig. 3). Furthermore, the leave-one-out approach, as illustrated in Fig. 4, affirmed the robustness and stability of the MR analysis, demonstrating that the exclusion of any SNP did not significantly impact the overall findings, supporting the reliability and stability of the MR analysis.

**Table 2:** Sensitivity analysis causality from blood metabolites on SLE.

Metabolites	Q_pval (IVW)	MRegger_interpreter	MRegger_interpreter_pval	MRPRESSO_GLOBAL
Carbohydrate				
1,5-Anhydroglucitol (1,5-AG)	3.91E−09	0.085572857	0.058921943	< 0.001
Erythrose	0.223640701	0.040746711	0.594611092	0.274
Lipid				
5-Dodecenoate (12:1n7)	0.225006148	−0.056271325	0.216472562	0.178
1-Linoleoylglycerophosphoethanolamine	0.989140627	0.021472062	0.438164124	0.963
Adrenate (22:4n6)	0.274124194	0.001703907	0.976979995	0.328
Amino acid				
Gamma-glutamylthreonine	0.366118463	0.148486033	0.042765681	0.413
Unknown				
X-10810	0.989140627	0.021472062	0.438164124	0.963
X-11412	0.458807845	−0.010865033	0.641879865	0.471
X-11905	0.894506947	−0.02809744	0.357888134	0.917
X-12038	0.631738147	0.07242924	0.109834223	0.657
X-12217	0.441095877	−0.08379171	0.128447555	0.466
X-06351	0.718786965	0.06606423	0.322291501	0.684

## Discussion

Our study aimed to explore the potential causal effects of 486 blood metabolites, using genetic proxies, on SLE development. Integrating two large-scale GWAS datasets and employing rigorous MR design, our analyses highlighted metabolites genetically associated with reduced SLE risk. These metabolites include X-06351, 1,5-anhydroglucitol (1,5-AG), X-11905, gamma-glutamylthreonine*, linoleoylglycerophosphoethanolamine *, X-12038, X-12217 and 5-dodecenoate (12:1n7). Elevated levels of these metabolites were associated with a lower risk of developing SLE. Conversely, we also found that genetic susceptibility to increased levels of certain metabolites, namely erythrose, X-10810, 1-, X-11412, adrenate (22:4 n6), was associated with a higher risk of SLE.

SLE manifests as a multifactorial autoimmune disease characterised by self-tolerance loss, autoantibody production and immune complex-related damage across multiple tissues and organs such as the kidneys, skin, joints, blood and nervous system^26^. Its clinical manifestations vary from mild mucocutaneous manifestations to life-threatening renal, nervous system, or multi-organ involvement^27^. Despite the association between genetic, hormonal, and environmental factors and diverse clinical SLE manifestations, its precise pathogenesis remains unclear. Early diagnosis is crucial to prevent irreversible organ damage, underscoring the need for reliable lupus-associated organ damage biomarkers to optimise SLE treatment. Metabolomics technologies have sparked interest in exploring the potential significance of metabolites in SLE. Blood metabolites can reflect both endogenous and exogenous processes, providing intuitive biological mechanism insights. For example, studies have reported that GC/MS tested serum from healthy controls and patients with SLE revealed that patients. The authors determined that nephritis patients can be distinguished from SLE patients by detecting elevated levels of serum lipid metabolism and decreased acetate levels ^28,29^. Although existing literature provides conflicting results on some metabolites, apparent serum metabolite differences between patients with SLE and healthy controls do exist. Most studies showed an increase in fatty acids and a decrease in most amino and organic acids, while others reported that oxidative stress was increased in SLE, especially in the form of decreased intracellular antioxidant glutathione^30^. Considering that these metabolome changes in the blood reflect intracellular changes, a more precise study of cellular metabolic regulation of immune cell subsets in SLE is required. Although the metabolites' association with SLE is strongly indicated, further research is needed to establish a clear causal link and better understand the underlying mechanisms. Such investigations will enhance our knowledge of SLE pathogenesis and potentially aid in developing effective strategies for early detection and prevention in the future. Therefore, we conducted a pivotal MR study to clarify the causal relationship between blood metabolites and SLE, offering new avenues for SLE screening and treatment.

In addition to the unknown blood metabolites, this MR study identified four blood metabolites (1,5-anhydroglucitol (1,5-AG), 1-linoleoylglycerophosphoethanolamine *, gamma-glutamylthreonine and 5-dodecenoate (12:1n7)) deemed protective against SLE. Although limited reports discuss their effects, certain insights exist, warranting further investigations. For instance, 1, 5-anhydrous glucose reflects blood glucose levels over 1–2 weeks and may exhibit antioxidant properties concerning type 1 diabetes, combatting cellular damage and inflammatory responses by reducing oxidative stress and free radical production^31^. This could be related to glucose metabolism and glucose ketone group regulation. Gamma-glutamylthreonine (γ-glutamyl-threonine) has an amino acid structure similar to glutamine and is synthesised from glutamate and threonine, a cystine precursor, through the amino acid metabolic pathway. As a compound containing γ-glutamylthreonine residues, γ-glutamylthreonine may also have antioxidant properties. Notably, γ-glutamyl residues are involved in synthesising glutathione (GSH), an important antioxidant that protects cells from damage by reducing intracellular oxidative stress responses and scavenging free radicals^32^. However, the specific functions and mechanisms of γ-glutamylthreonine remain incompletely understood., warranting further study. Similarly, 5-dodecenoate, also known as 12:1n7, is an Omega-3 fatty acid with potential health benefits. Omega-3 fatty acids have been demonstrated to reduce the activity of SLE^33^, affecting the production of many inflammatory proteins, including cytokines and adhesion molecules. Omega-3 fatty acids also reduce the production of pro-inflammatory cytokines (Tnf-α, Il-1β, and IL-6) in response to LPS but increase the concentrations of the anti-inflammatory cytokine IL-10 ^10^. Several studies using EPA and DHA as supplements in healthy human volunteers have reported reduced TNF, IL-1 and IL-6 production by LPS-stimulated monocytes or monocyte^34–37^. Similarly, human trials have demonstrated the benefits of oral n-3 fatty acids in rheumatoid arthritis and in stabilising advanced atherosclerotic plaques^12,38–40^. Furthermore, the intravenous administration of n-3 fatty acids may benefit critically ill patients by reducing inflammation. Our findings indicate an inverse correlation between 5-dodecenoate abundance and SLE incidence, suggesting a potential therapeutic approach targeting this taxon for SLE management.

Furthermore, our study highlighted that elevated erythrose and adrenate (22:4 n6) levels increased the risk of SLE. Adrenate (22:4 n6), a long-chain Omega-6 fatty acid, is involved in lipid metabolism and shares associations with inflammatory processes. Many studies have shown that the omega-6 polyunsaturated fatty acid arachidonic acid (ARA) exerts an essential effect on fatty acids in cell membrane phospholipids involved in inflammation. ARA released from cell membrane phospholipids acts as a substrate for cyclooxygenase (COX), lipoxygenase (LOX) and cytochrome P450 enzymes to produce eicosanoid family mediators, resulting in high ARA content being directly related to inflammation. Moreover, eicosanoid acids are essential regulators and mediators of inflammatory processes and include prostaglandins (PGs), thromboxanes and leukotrienes (LTs) ^41–43^. Inflammatory stimuli can upregulate eicosanoid synthesis through enzymatic activation (e.g., release of ARA from membrane phospholipids by phospholipase A2) and enzyme-encoding gene expression. Many anti-inflammatory therapies, such as non-steroidal anti-inflammatory drugs and COX inhibitors, target ARA metabolism, suggesting that ARA metabolism is closely related to inflammatory processes. Erythrose, a four-carbon ketose sugar, is one of the simplest sugars in nature. Erythrose is not commonly found in free form but is an intermediate in several metabolic pathways. It is involved in the glycolysis process and contributes to acute T cell metabolic shifts towards aerobic glycolysis, a process observed in SLE CD4+ T cells. Moreover, ATP production mainly depends on OXPHOS^44^.Interestingly, healthy effector CD4+ T cells expanded in SLE patients, and naive CD4+ T cells in lupus-susceptible mice showed elevated glycolytic pathways and oxidative phosphorylation^8^. These results suggest that the elevation of the glycolytic pathway and OXPHOS in SLE CD4+ T cells is not a secondary effect of autoantibody exposure or differentiation, but rather an incidental effect associated with SLE pathogenicity. However, reports on the immune metabolism of B cells in SLE are scarce. Transgenic mice overexpressing B cell activating factors shift the metabolic state of B cells to the glycolytic pathway^45^. Unlike T cell receptor stimulation, which primarily activates the glycolytic pathway, B cell receptor (BCR) stimulation enhances both the glycolytic and oxidative phosphorylation pathways, indicating an elevated state of the glycolytic pathway in immune cells in SLE. Similarly, our results revealed that the abundance of the glycolytic intermediate metabolite Erythrose was positively correlated with SLE, implying that this taxon may serve as a novel therapeutic target. However, its specific role needs to be further explored.

There are several notable advantages to this MR analysis. To the best of our knowledge, this study stands out for its comprehensiveness in examining 486 blood metabolites and their potential impact on SLE. Rigorous methods were employed to address potential pitfalls, ensuring robust and reliable results. Various methods were implemented to ensure that factors violating the MR assumption were appropriately addressed and removed from the analysis. The study's commitment to producing convincing estimates is evident through their meticulous handling of potential confounding factors. By applying stringent methods and considering various factors, the researchers were able to generate robust and reliable results. Furthermore, the consistency across different MR methods further enhances the reliability of our findings. Additionally, sensitivity analyses reinforced the validity of the results under various conditions and assumptions.

However, our study also has its limitations. The availability of a restricted set of SNPs for comprehensive genome-wide exposure analysis poses a constraint, despite the satisfactory robustness observed in the selected SNPs’ F-statistics. Furthermore, limiting the analysis to individuals of European ancestry may restrict the findings’ generalisability, warranting validation in diverse ethnic groups. Additionally, expanding the sample size in future studies could enhance result reliability and accuracy of causal effect estimations.

## Conclusion

This MR study established a causal relationship between blood metabolites and SLE using genetic proxies. Additionally, the research identifies six distinct blood metabolites potentially associated with the development of SLE. These findings offer important insights into potential strategies for early screening, prevention and treatment of SLE, offering a roadmap for future clinical investigations in this domain. Furthermore, the MR analysis framework employed in this study serves as a valuable model for further explorations into the underlying aetiology and mechanisms driving SLE.

### Supplementary Information

Supplementary Table S1.

Supplementary Table S2.

Supplementary Table S3.

Supplementary Table S4.

## Data Availability

We have annotated the article with the source of all original data, please contact the original authors for access if needed. The results of this study can be obtained by contacting the corresponding author.
